# Antioxidant Properties of Cerium Oxide Nanoparticles Prevent Retinal Neovascular Alterations In Vitro and In Vivo

**DOI:** 10.3390/antiox11061133

**Published:** 2022-06-09

**Authors:** Annamaria Tisi, Fanny Pulcini, Giulia Carozza, Vincenzo Mattei, Vincenzo Flati, Maurizio Passacantando, Cinzia Antognelli, Rita Maccarone, Simona Delle Monache

**Affiliations:** 1 Department of Biotechnological and Applied Clinical Sciences, University of L’Aquila, 67100 L’Aquila, Italy; annamaria.tisi@univaq.it (A.T.); fanny.pulcini@graduate.univaq.it (F.P.); giulia.carozza@graduate.univaq.it (G.C.); vincenzo.flati@univaq.it (V.F.); simona.dellemonache@univaq.it (S.D.M.); 2 Biomedicine and Advanced Technologies Rieti Center, Sabina Universitas, 02100 Rieti, Italy; v.mattei@sabinauniversitas.it; 3 Department of Physical and Chemical Sciences, University of L’Aquila, 67100 L’Aquila, Italy; maurizio.passacantando@univaq.it; 4 Department of Medicine & Surgery, Bioscience and Medical Embryology Division, University of Perugia, 06129 Perugia, Italy

**Keywords:** oxidative stress, wet AMD, VEGF, RPE, cerium oxide nanoparticles, ARPE-19, HUVEC, angiogenesis, glycative stress

## Abstract

In this study, we investigated whether cerium oxide nanoparticles (CeO_2_-NPs), a promising antioxidant nanomaterial, may contrast retinal vascular alterations induced by oxidative damage in vitro and in vivo. For the in vivo experiments, the light damage (LD) animal model of Age-Related Macular Degeneration (AMD) was used and the CeO_2_-NPs were intravitreally injected. CeO_2_-NPs significantly decreased vascular endothelial growth factor (VEGF) protein levels, reduced neovascularization in the deep retinal plexus, and inhibited choroidal sprouting into the photoreceptor layer. The in vitro experiments were performed on human retinal pigment epithelial (ARPE-19) cells challenged with H_2_O_2_; we demonstrated that CeO_2_-NPs reverted H_2_O_2_-induced oxidative stress-dependent effects on this cell model. We further investigated the RPE–endothelial cells interaction under oxidative stress conditions in the presence or absence of CeO_2_-NPs through two experimental paradigms: (i) treatment of human umbilical vein endothelial cells (HUVECs) with conditioned media from ARPE-19 cells, and (ii) coculture of ARPE-19 and HUVECs. In both experimental conditions, CeO_2_-NPs were able to revert the detrimental effect of H_2_O_2_ on angiogenesis in vitro by realigning the level of tubule formation to that of the control. Altogether, our results indicate, for the first time, that CeO_2_-NPs can counteract retinal neovascularization and may be a new therapeutic strategy for the treatment of wet AMD.

## 1. Introduction

Age-related macular degeneration (AMD) leads to the degeneration of retinal pigment epithelium (RPE) cells and of the underlying light-detecting photoreceptor (PR) cells. Such forms of retinopathies are expected to become the leading cause of blindness and vision impairment in humans. Currently, effective treatments to prevent RPE and PR cell death are missing [1]. Two major forms of AMD exist: (i) dry (or non-exudative) AMD, accounting for about 90% of AMD patients, and (ii) wet (or exudative) AMD, accounting for about 10% of AMD patients [2]. Although less frequent, wet AMD represents the worst pathological scenario compared to dry AMD, since it progresses more rapidly and with a more aggressive outcome, characterized by choroidal neovascularization (CNV), subsequent blood-retinal barrier (BRB) breakdown, and retinal degeneration [3]. Vascular endothelial growth factor (VEGF) has been recognized as one of the main factors involved in wet AMD development, being up-regulated in affected patients [1,3]. Accordingly, anti-VEGF-based therapies are considered the gold standard treatment for wet AMD, preventing the development of new vessels and delaying the progression of the disease [4]. Nonetheless, this therapeutic approach has several shortcomings. First, multiple intravitreal injections are needed to guarantee the protective effect over time; this may cause retinal detachment, infections due to repeated surgical procedures, and patient discomfort. Secondly, but not less importantly, some patients are insensitive to anti-VEGF therapy, indicating that other processes may drive the development of the disease in addition to VEGF up-regulation [5].

Indeed, the pathogenesis of AMD is not fully understood since it is a multifactorial disease. In this context, oxidative stress is considered one of the major risk factors in the pathogenesis of AMD, together with aging, cigarette smoking, high fat diet, light exposure, and some genetic polymorphisms [6]. The retina is constantly exposed to mild levels of oxidative stress because of environmental light exposure and the high rate of metabolism of this tissue, which causes the production of reactive oxygen species (ROS). RPE cells, the main constituents of the blood retinal barrier (BRB), are particularly subjected to oxidative stress and this impairs PR cell function. In fact, one of the RPE cell roles is the continuous renewal of the external segments of photoreceptors, together with the restoration of essential molecules involved in the visual cycle [6]. Nevertheless, RPE, along with the entire retina, acts as an endogenous antioxidant system that regulates the equilibrium with free radicals in the tissue [7]. With aging, this auto-regulation mechanism is impaired and, together with the parallel accumulation of toxic metabolites, leads to a vicious cycle with progressive oxidative stress burden [8]. Moreover, much evidence indicates that increased oxidative stress induces the upregulation of VEGF expression in RPE cells [9,10]. This leads to: (i) RPE dysfunction, characterized by epithelial–mesenchymal transition (EMT), which culminates in the loss of the RPE phenotype [11]; (ii) a breakdown of the BRB, due to the disruption of RPE tight junctions and subsequent loss of cell-cell adhesion and polarization of RPE cells [11]; and (iii) choroidal neovascularization (CNV), characterized by the proliferation of new vessels arising from the choroid to the neuroretina [12].

In our previous studies, we tested a new nanotechnological approach for the treatment of AMD, based on CeO_2_-NPs. These nanoparticles (NPs) show peculiar antioxidant properties with an auto-regenerative radical scavenging activity, which allows a prolonged efficacy [13]. CeO_2_-NPs showed protective effects in in vitro and in vivo models of AMD, by counteracting multiple aspects of AMD, such as: RPE dysfunction and death [14], photoreceptor death [15,16], microglia activation and inflammation [17], autophagy alterations [14], and the accumulation of auto-fluorescent deposits [18]. To date, the possible protective effect of CeO_2_-NPs against retinal pathological neovascular events has never been investigated. The authors investigated the protective effects of CeO_2_-NPs against the oxidative stress that induces RPE dysfunction and consequent choroidal neovascularization, two characteristic aspects of wet AMD.

Specifically, we set out to achieve the following aims:

(1) To investigate whether intravitreal injection of CeO_2_-NPs is able to modulate the VEGF expression and to counteract neovascularization in an in vivo model of AMD.
(2) To highlight the antioxidant effects of CeO_2_-NPs on ARPE-19 cells by analyzing the modulation of specific oxidative stress-related markers.
(3) To understand whether, in an in vitro model, the ARPE-19 dysfunction induced by oxidative stress could promote neovascularization in HUVEC cells and if it might be prevented by CeO_2_-NPs treatment.

## 2. Materials and Methods

### 2.1. In Vivo Experimental Design

All in vivo experiments were performed according to the ARVO statement for the use of animals in ophthalmic and vision research. All experiments were approved by the Italian Ministry of Health, authorization number 763/2020-PR, approved in July 2020.

Sixteen Sprague Dawley (SD) albino rats were born and raised in dim cyclic light condition (12 h light, 12 h dark) with an ambient light level of approximately 5 lux and food and water ad libitum. The animals were exposed to high intensity light for 24 h and euthanized one week thereafter in order to induce retinal damage and neovascularization, as previously reported [19]. A group of animals received intravitreal injection of CeO_2_-NPs three days before light exposure (CeO_2_-NPs-LD) and it was compared to untreated light damaged animals (LD) and healthy animals (CTRL). Electroretinogram recordings (ERG) were performed before starting the experiment in order to assess physiological visual function, as previously described [19]. To assess retinal protection by CeO_2_-NPs, ERG was also performed at the end of the experiment, confirming a significant functional preservation [15,16].

**Figure 1:**
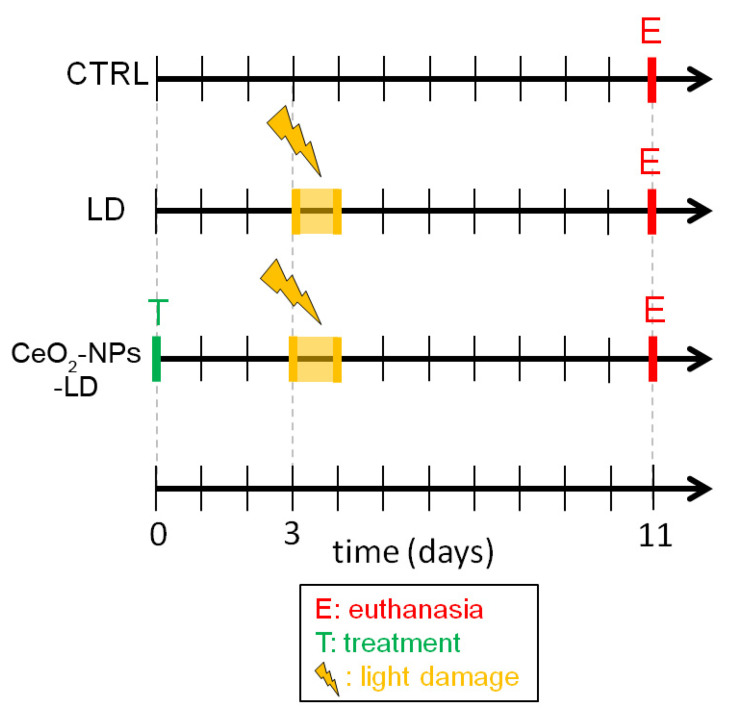
Experimental design of the in vivo study. The in vivo experiments were performed on Sprague Dawley albino rats divided in three experimental groups (*N* = 4/group). (1) CTRL: control group of animals without retinal damage or treatment; (2) light damage (LD): animals subjected to light damage for 24 h and then euthanized seven days thereafter; (3) CeO_2_-NPs-LD: animals treated with cerium oxide nanoparticles via intravitreal injection, then subjected to light damage for 24 h and euthanized seven days later.

In summary, the animals were divided into three experimental groups: (1) CTRL, (2) LD, and (3) CeO_2_-NPs-LD, as reported in Figure 1. Additionally, a group of healthy animals received intravitreal injection of CeO_2_-NPs (CeO_2_-NPs -CTRL) in order to verify a possible modulation of VEGFA by the treatment.

### 2.2. Intravitreal Injections of CeO_2_-NPs

CeO_2_-NPs were synthesized using a mixture of Ce(NO_3_)_3_·6H_2_O and ethylene glycol was stirred for 30 min. Ammonium hydroxide (NH_4_OH) was then added and the resulting product was calcined in an air furnace at 500 °C. The detailed synthesis procedure and the structural and electronic properties of CeO_2_-NPs are reported in Passacantando and Santucci, 2013 [13].

CeO_2_-NPs were administered through intravitreal injection in both eyes, according to a previously published method [14,15]. The treatment was performed three days before LD, that is, a condition which allows retinal protection in our model, as previously reported [14,16,20]. Therefore, we repeated the same experimental condition in order to investigate whether retinal protection by CeO_2_-NPs is also associated with any anti-neovascular effects. Before the surgery, the rats were anesthetized with an intraperitoneal injection of Ketamine/Xylazine (10 mg/100 g–1.2 mg/100 g). An amount of 2 μL of CeO_2_-NPs (1 mM in NaCl 0.9%) were intravitreally injected using a Hamilton syringe under total sterile conditions. To avoid post-surgical infection, a drop of ophthalmic antibiotic (Tobral 0.3%) was applied to the site of the injection. The animals were then returned to their cages, monitored to ensure complete awakening and good health, and then housed in the animal room in dim cyclic conditions until light damage.

### 2.3. In Vivo Retinal Light Damage

Acute retinal light damage was performed as reported in our previous studies [14,18,19]. Briefly, the animals were dark-adapted overnight, then placed in individual plexiglass cages with food and water and positioned in the light damage apparatus. Light exposure (1000 lux) started at 9 a.m.—in order to not interfere with the circadian rhythm of the animals—and lasted 24 h. At the end of the light damage session, all animals were returned to dim cyclic light conditions (5 lux) for seven days and then euthanized for morphological and molecular analyses. The retinas were analyzed seven days after the light injury, since we previously demonstrated that the major light induced-neovascular events occur in the retina seven days after light exposure [19].

### 2.4. Retinal Samples Collection

At the end point, all animals were euthanized and retinal samples were collected. Specifically, one eye from each animal was analyzed using the Western blot technique, while the contralateral was collected for morphological analysis. The eyes were enucleated and the cornea, lens, and vitreous were removed. Afterwards, the retina was gently isolated from the eye cup. The whole procedure was conducted under a stereomicroscope and by keeping the samples on ice to avoid tissue degradation. The retinas used for Western blot analysis were immediately frozen and stored at a temperature of −80 °C, while the ones used for whole mounts procedure were immediately fixed in paraformaldehyde. The processing of the retinas for each technique is detailed in dedicated paragraphs below.

### 2.5. Western Blot Analysis

For Western blot analysis of in vivo samples, total proteins were extracted from rat retinas by using a Dounce Homogenizer and a lysis buffer (50 mM Tris-HCl pH 7.5, 1% Triton X-100, 0.1% SDS, ethylenediaminetetraacetic acid EDTA 5mM, Halt Protease and Phosphatase Inhibitor Cocktail from Thermo Fisher Scientific Inc. (Monza, Italy) and QS dH_2_O). Bradford Assay (Bio-Rad Laboratories, Milan, Italy) was used to quantify the protein content, and 70 µg of the proteins were run on a Bolt 4–12% Bis-Tris Plus (Thermo Fisher Scientific) at 200 V for 20 min. The proteins were then transferred to the PVDF membrane (Thermo Fisher Scientific) through an iBlot 2 Dry Blotting System (Invitrogen, Waltham, MA, USA, IB21001).

For quantification of VEGF, non-specific bindings were blocked with 5% non-fat dry milk in TBST (Tris-buffered saline with 0.1% Tween) at RT for 1 h. Afterwards, the membranes were incubated with primary antibody anti-VEGF (Abcam #ab46154) (1:200) diluted in 5% non-fat dry milk in TBST overnight at 4 °C, and then incubated with the specific anti-rabbit horseradish peroxidase (HRP)-conjugated secondary antibody (1:2000 in 1% non-fat dry milk in TBST) for 1 h at room temperature (RT). The bands were detected by incubating the membranes in SuperSignal West Pico Plus (Thermo Fisher Scientific Inc., Monza, Italy) chemiluminescent substrate and using a ChemiDoc XRSplus imaging system (Bio-Rad Laboratories, Milano, Italy). The optical densities of blot bands were obtained by ImageJ (U.S. National Institutes of Health, Bethesda, MA, USA) software and were normalized versus α-tubulin as loading control.

### 2.6. Isolectin Staining and Vasculature Analysis of Rat Retinas

**Figure 2:**
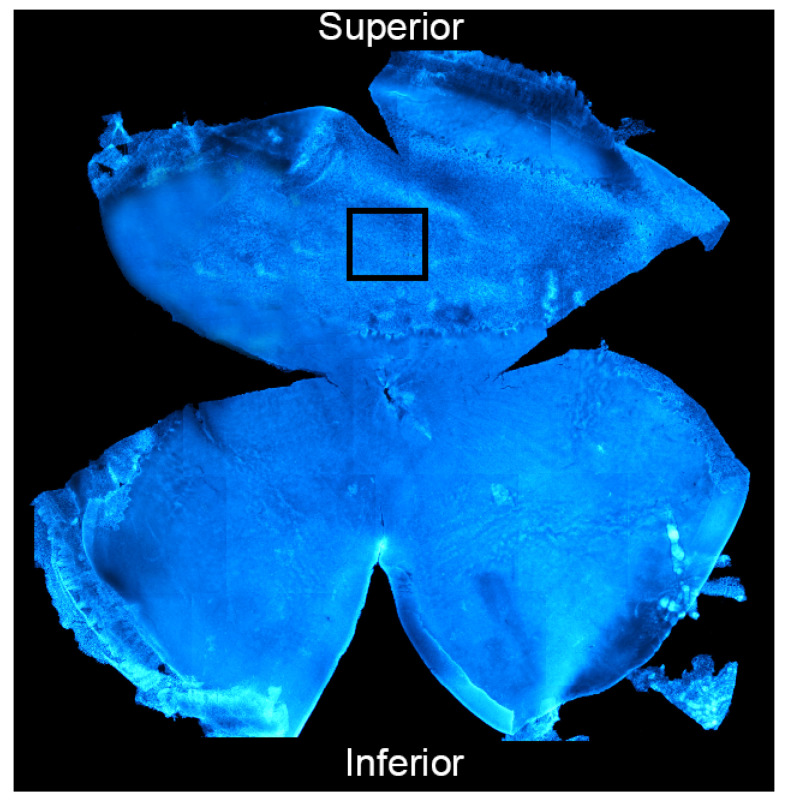
Representative image of a whole mounted retina. The image was obtained by reconstruction from multiple images acquired with a fluorescence microscope; the retina was stained with bisbenzimide nuclear dye (blue). The black frame highlights the central superior retina, that is, the area selected for the analysis of the retinal vasculature.

In order to investigate the retinal vasculature, retinas were isolated from the eye, fixed in 4% paraformaldehyde for 1 h at RT and washed with 0.1 M TrisHCl (pH 7.4). Afterwards, isolectin B4 staining was performed to visualize the vessels on whole mounted retinas. Specifically, 10% goat serum (GS) was used to block non-specific binding sites and the retinas were then incubated with Isolectin B4 Alexa Fluor dye conjugates (1:150 in 1% GS) for 36 h at 4 °C. The samples were then counterstained with the nuclear staining bisbenzimide in order to identify the different retinal layers. In order to obtain flat retinas and to mount them on gelatine and poly-l-lysine-coated slides, four small radial cuts were made starting from the edge towards the center of the retina with surgical scissors under a stereomicroscope. The retinas were then acquired by a Nikon Eclipse 80i (Melville, New York, NY, USA) confocal microscope. We focused our analysis on the deep retinal plexus (which lays in the outer plexiform layer, OPL) at the center of the superior retina (2.5 mm from the optic nerve), that is, the primary site of light-induced degeneration and vascular alterations known as “hot spot” [19]. An example of a whole mounted retina and the selected area for the analysis is shown in Figure 2. Neovascularization was then analyzed in terms of (i) “vessels percentage area” quantified through AngioTool software 0.6, and (ii) “number of tufts of neovascularization” counted on the acquired images and identified with a defined swelling morphology (at the terminals and in the middle of the vessels).

### 2.7. Cell Culture

The experiments were conducted on human retinal pigment epithelial (ARPE-19) cells (ATCC, Manassas, VA, USA). ARPE-19 cells were cultured in a medium containing Dulbecco’s Modified Eagle Medium (DMEM) and Ham-F12 Medium mixture (1:1) with 10% fetal bovine serum (FBS) (Corning, New York, NY, USA), 1% Glutamine, and 1% Penicillin/Streptomycin (Gibco, Thermo-Fisher Scientific, Monza, Italy) in a humidified atmosphere at 37 °C with 5% CO_2_. Cells were used between passage 4 and 15. Human umbilical vein endothelial cells (HUVECs) (Lonza, Walkersville, MD, USA) were cultured in Endothelial Growth Medium (EGM-2) (Lonza, Walkersville, MD, USA) obtained by adding specific endothelial growth factors to the Endothelial Basal Medium (EBM-2) (Lonza, Basel, Switzerland). For cell treatments, CeO_2_-NPs’ synthesis was performed as previously described [13]. HUVECs were used from passage 3 to 12. All in vitro experiments were performed in triplicate.

### 2.8. Oxidative Stress Induction and CeO_2_-NPs Treatment

To induce oxidative stress, ARPE-19 cells were exposed to increasing concentrations of H_2_O_2_ (0, 250, and 500 µM) for 24 h. Control cells were treated only with complete medium.

CeO_2_-NPs (0.1 mM) were added to the culture medium in the presence or absence of H_2_O_2_.

### 2.9. Preparation of Conditioned Media (CM) of ARPE-19 Cells

ARPE-19 cells were cultured under controlled conditions (37 °C in a humidified atmosphere and 5% CO_2_) and, upon reaching 70% confluence, they were detached from the culture plate, by washing with trypsin. The cells were plated in T-75 flasks at a concentration of 1 × 10^4^ cells/cm^2^ and left overnight in the incubator. ARPE-19 cells were treated with two concentrations of H_2_O_2_ (250 µM and 500 µM) in the presence and absence of CeO_2_-NPs. After 24 h from the treatment, complete culture medium was replaced with serum-free medium, and the cells were left in culture for a further 24 h. CM was collected and centrifuged at 260× *g* at 4 °C for 10 min to remove cell debris. The supernatant was stored at −80 °C until use for the experiments.

### 2.10. Enzyme Activity Measurements

#### 2.10.1. Superoxide Dismutase 2 (SOD 2) Activity Assay

SOD 2 activity was determined by the indirect xanthine/xanthine oxidase method [21] Xanthine oxidase oxidizes xanthine (0.05 mM) and produces a superoxide anion, which in turn reduces cytochrome C (0.01 mM). This reduction is spectrophotometrically followed by the increasing absorbance at 550 nm. When SOD 2 is added to the reaction mixture this subtracts the superoxide anion inhibiting the reduction of cytochrome C, thus causing a minor increase in absorbance. SOD 2 activity was expressed in terms of units/mg of protein, where 1 unit is defined as the amount of enzyme that inhibits the rate of cytochrome C reduction by 50%. The activity assay was carried out in triplicate at 25 °C, the temperature normally used in the xanthine oxidase assay method.

#### 2.10.2. Glutathione Peroxidase (GPx) Activity Assay

GPx activity was measured at pH 7.6 and 37 °C in the presence of 0.5 mM Glutathione (GSH). The assay mixture consisted of 0.12 mM NADPH, 0.5 mM GSH, 1 unit/mL of glutathione reductase, and 0.2 mM of H_2_O_2_. NADPH disappearance was monitored at a wavelength of 340 nm [22].

#### 2.10.3. Glyoxalase 1 (Glo1) Activity Assay

Glo1 is a cytoplasmic enzyme that prevents glycation reactions by using GSH as a cofactor [23]. To measure its activity, the cells were lysed in an extraction buffer containing 100 mM KH_2_PO_4_, 1.5 mM dithiothreitol (DTT), and 1 mM EDTA (pH 7). The cells were homogenized and centrifuged at 16,000× *g* for 30 min at 4 °C and the protein extracts were used for the measurement of the Glo1 enzyme activity. The assay mixture solution contained 0.1 M of sodium phosphate buffer pH 7.2, 2 mM of methylglyoxal (MG), and 1 mM of reduced GSH. The reaction was monitored spectrophotometrically by following the increase in absorbance at 240 nm and 25 °C. A unit of activity was defined as 1 µmol SD lactoylglutathione produced per minute.

#### 2.10.4. Glutathione-S-Transferase (GST) Activity Assay

GST activity was analyzed using a mixture of 0.1 M potassium phosphate, 1 mM EDTA, 1 mM 1-chloro-2,4-dinitrobenzene (CDNB), and 2 mM GSH at pH 6.5 and 25 °C. CDNB conjugation was recorded at 340 nm as described by Habig and Jakoby [24]. For data analysis, an enzyme unit was defined as the amount of enzyme that catalyzes its specific reaction at the rate of 1 μmol of substrate/min at the saturation concentration of the substrate. The results were calculated as μmol of the specific enzyme per mg of protein and were performed in triplicate.

#### 2.10.5. Glutathione (GSH) Assay

GSH was measured by using the GSH assay kit (colorimetric) from Bio Vision Inc. (Milpitas, CA, USA) according to the manufacturer’s instructions. Briefly, the assay is based on an enzymatic cycling method in the presence of GSH and chromophore. The reduction of the chromophore produces a stable product, which can be tracked at 450 nm. Its absorbance is directly proportional to the GSH content in the sample.

### 2.11. Heme Oxygenase-1 (HO-1) Detection

HO-1 is the enzyme that catabolizes heme. Its activity is cleaving the heme ring to form biliverdin. The HO-1 protein level was measured by using a specific, commercially available enzyme-linked immunosorbent assay (ELISA) kit from Abcam (Prodotti Gianni, Milan, Italy) according to the manufacturer’s instructions. Briefly, 50 µL of standard and samples were added to appropriate wells along with 50 µL of Antibody Cocktail. The plate was incubated for 1 h at RT in a shaker. Following several wash steps, all wells were incubated with 100 µL of tetramethylbenzidine (TMB) substrate for 15 min in the dark and then with 100 µL of Stop Solution. The reading was performed at 450 nm with a Mindray MR-96A Microplate Reader (Mindray Medical Italy S.r.l., Milan, Italy).

### 2.12. 5-Hydro-5-Methylimidazolone (MG-H1) Protein Adducts Detection

The MG-H1 protein adducts were measured using a competitive ELISA kit from Cell Biolabs Inc. (cat. STA-811, DBA Italia S.r.l.) according to the manufacturer’s instructions. The procedure has already been reported by Delle Monache et al. 2021 [25]. Briefly we pre-coated the MG conjugate on the ELISA plate. Then, samples or MG-BSA (Bovin Serum Albumin) standards were added in triplicate and a specific anti-MG monoclonal antibody was incubated for 1 h at RT. After washing the plate, it was incubated with a horseradish peroxidase (HRP) conjugated secondary antibody. The content of MG-H1 adducts in the protein samples was determined through a 4P logistic regression equation by measuring the absorbance at 450 nm with a Mindray MR-96A microplate reader (Mindray Medical Italy S.r.l., Milan, Italy).

### 2.13. Malondialdehyde (MDA) Detection

The intracellular concentration of MDA was determined according to the method described by Draper et al. [26] with some modifications. In particular, the cell homogenate was mixed with butylated hydroxytoluene (BHT) at 500 ppm in methanol and with 10% trichloroacetic acid (TCA). The resulting mixture was boiled for 30 min and then it was centrifuged at 1500× *g* for 10 min. The supernatant was mixed with a solution of 2-thiobarbituric acid (TBA) and boiled again for 30 min. After cooling to RT, 500 μL of each sample was extracted with 1 ml of n-butanol using a vortex mixer and then centrifuged at 1000× *g* for 5 min. The top layer was collected and filtered through a 0.2 µm Whatman Puradisc syringe filter, followed by analysis with the PerkinElmer HPLC system (PerkinElmer Inc., Wellesley, MA, USA) and a 5 µm Reliasil C18 column (Column Engineering, Ontario, CA, USA). Excitation and emission wavelengths were set at 515 nm and 550 nm, respectively.

### 2.14. Phalloidin Staining

To visualize the cellular cytoskeleton, cells were stained with phalloidin. Briefly, cells were seeded in 6-well plates at 2 × 10^5^/well density, and grown for 24 h. Afterwards, cells were treated with H_2_O_2_ (250 µM and 500 µM) in the presence or absence of CeO_2_-NPs (0.1 mM). At the end of the experiment, the cells were fixed with 4% PFA (Paraformaldehyde) for 10 min. Non-specific binding sites were blocked with 3% BSA and 0.1% Triton for 30 min, and then the ARPE-19 cells were labeled with FITC-Phalloidin (Fluorescein Isothiocyanate) (Sigma Aldrich, Saint Louis, MO, USA) (1:250 in PSB 1X) for 40 min at RT and then washed three times with PBS. Finally, nuclei were counterstained with bisbenzimide nuclear dye and the images were acquired with a Floid Cell Imaging Station (Thermo Fisher Scientific). Cell area was then calculated by performing manual segmentation of single cells on the acquired images through the Image J software 1.8.0 (U.S. National Institutes of Health, Bethesda, MD, USA). For each experimental condition, ~200 cells were measured for robust statistical analysis.

### 2.15. VEGF Determination

Human VEGF were detected in all ARPE-19 CM using a competitive ELISA kit (Euroclone, Milan, Italy) following the manufacturer’s instructions. For each sample, cells were recovered and total protein content was determined. The absorbance was measured at 450 nm using an ELISA microplate reader (TECAN) and the values normalized with respect to the cell number. Experiments were performed in triplicate and repeated 3 times. VEGF was within the dynamic range of the ELISA standard curve and calculated as pg/mL/g of protein.

### 2.16. In Vitro Tubule-Like Formation

The ECMatrix assay kit (Chemicon, Millipore, USA) was used to study tubule formation in vitro according to the manufacturer’s instructions. For the test, 15-well microslides (IBIDI Munich, Germany) were coated with 10 μL of Matrigel/well and left to solidify at 37 °C for 30 min. A total of 1.5 × 10^4^ HUVECs for each well were seeded and a CM (diluted 1:4) derived from ARPE-19 was added. DMEM-F12 was used as the control. The micro-slide was then incubated, under controlled conditions, for up to 12–16 h. The degree of angiogenic response was assessed by acquiring images of each well using a Nikon inverted phase contrast microscope. The quantitative analysis was obtained through Image J software [27], considering the branching index, that is, the number of junctions formed for each field. In addition, mean values and standard deviation (SD) were determined for each analysis. Three independent experiments were performed for each treatment.

### 2.17. Cell Viability Assay

The effect of H_2_O_2_ on endothelial cell proliferation was evaluated by seeding the cells in a 96-well plate at a density of 5 × 10^3^ cells/well. HUVECs were maintained in a 37 °C incubator in a humidified atmosphere containing 5% CO_2_ for 24 h. Thereafter, the cells were treated with the various concentrations of H_2_O_2_ (100 µM, 200 µM, 300 µM, 400 µM, 500 µM, and 600 µM) for 24 h. To assess cell viability, the HUVECs were fixed with 4% PFA, stained with crystal violet solution (1%), and solubilized as described above.

### 2.18. Tubule Formation in Co-Culture Assay

To evaluate the direct effect that ARPE-19 treated with H_2_O_2_ and CeO_2_-NPs could have on tubule formation by HUVECs, a co-culture modified tubule formation assay was performed using Culture-Insert 2 Well in 35 mm µ-Dish (IBIDI Munich, Germany).

Briefly, 5.5 × 10^4^ ARPE-19 per µ-Dish were seeded, outside the insert, kept in a humidified incubator at 37 °C and 5% CO_2_ for 24 h and then treated with the various combinations of H_2_O_2_ (250 µM and 500 µM) and CeO_2_-NPs (0.1 mM). After 24 h, the two chambers of insert were coated with 35 µL of Matrigel (Chemicon, Millipore) and left in the incubator for about 1 h. A total of 4 × 10^4^ HUVECs for each chamber were seeded and incubated again for 1 h. Then, 5 ml of medium (2.5 mL EGM-2 and 2.5 mL DMEM-F12) were added in each µ-Dish to allow the exchange of factors between ARPE-19 (externally) and HUVEC (internally). After 16 h, all chambers of insert were photographed and tubule formation was assessed. Data analysis was performed as explained in the previous section (In Vitro Tubule-Like Formation).

### 2.19. Statistical Analysis

The statistical analyses were performed through one-way ANOVA test and first type error was set at 5%. Post hoc comparisons were conducted using Tukey’s test. The statistical analysis was conducted using SigmaPlot 12.0 software.

## 3. Results

### 3.1. Effects of CeO_2_-NPs on Retinal Vasculature In Vivo

We have previously demonstrated that the retinal light damage (LD) model mimics the main features of wet AMD, leading to the infiltration of new vessels into the photoreceptor layer, the increase in tuft numbers, and up-regulation of VEGFA [19]. On this basis, we used the LD model to investigate the potential protective effect of intravitreally injected CeO_2_-NPs against retinal vascular alterations.

**Figure 3:**
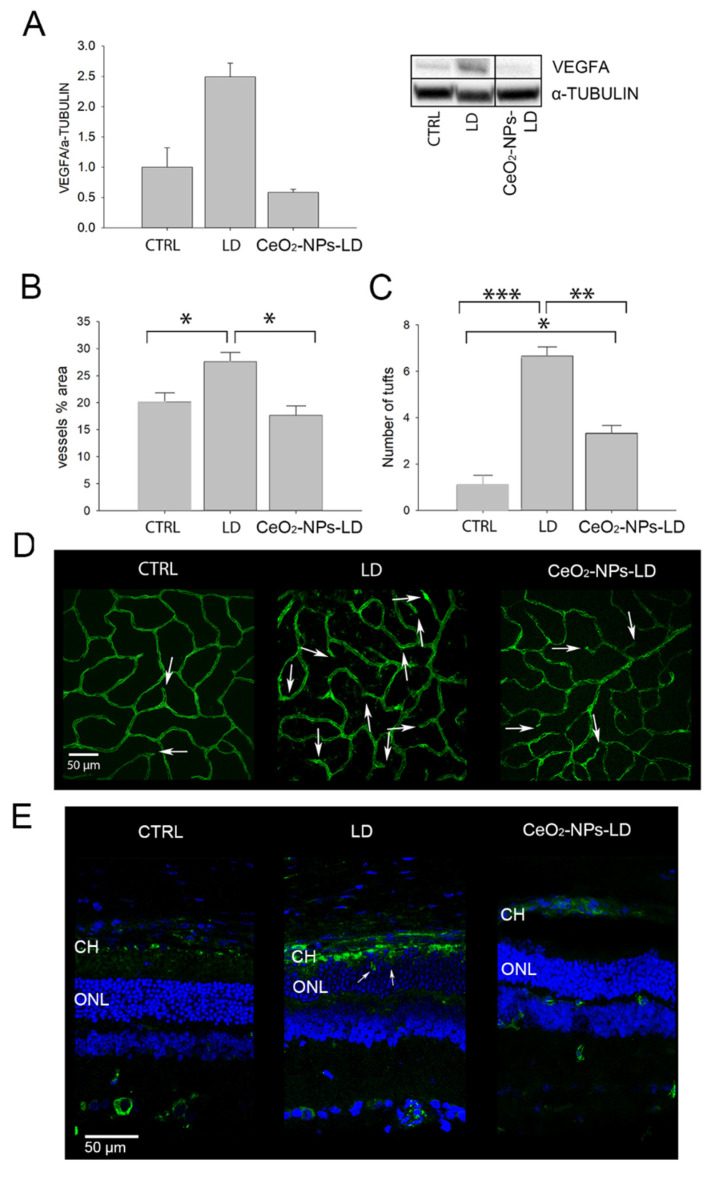
Intravitreal injection of CeO_2_-NPs prevents neovascularization in the LD animal model. (**A**) Western blot analysis of VEGFA in CTRL, LD, and CeO_2_-NPS-LD groups (*n* = 4). The graph shown as the mean ± SE (standard error) of VEGFA (folds vs. CTRL) normalized versus α-Tubulin. Statistical analysis: one-way ANOVA followed by Tukey’s test, ** *p* < 0.01. Entire Western blot bands are shown in Supplementary Figure S2. (**B**,**C**) Vessel analysis of the deep retinal plexus. (**B**) Vessels percentage area and (**C**) number of tufts of neovascularization. Graphs are shown as mean ± SE (*n* = 4). Statistical analysis was performed by one-way ANOVA followed by Tukey’s test; * *p* < 0.05, ** *p* < 0.01, *** *p* < 0.001. (**D**) Representative confocal images of whole mounted retinas stained with Isolectin B4 (green); the white arrows indicate the tufts; scale bar: 50 µm. (**E**) Representative confocal images of retinal cryosections stained with Isolectin B4 (green) and counterstained with bisbenzimide nuclear dye (blue). The white arrows indicate the vessels sprouting from the choroid into the Outer Nuclear Layer (ONL); scale bar: 50 µm. CTRL: control; LD: rats exposed to high intensity light for 24 h and euthanized seven days thereafter; CeO_2_-NPs-LD: rats treated with intravitreal injection of cerium oxide nanoparticles 3 days before light damage and euthanized seven days thereafter.

First, we investigated the protein levels of VEGFA and found that it was significantly reduced in the retinas of the CeO_2_-NPs-LD group compared to the LD group (Figure 3A). Moreover, CeO_2_-NPs did not induce any changes in VEGFA expression when injected in healthy animals (CeO_2_-NPs-CTRL) compared to CTRL (Figure S1).

Afterwards, we investigated whether CeO_2_-NPs could also prevent neovascularization of the retinal vasculature and the infiltration of new vessels from the choroid to the photoreceptor layer. Since CeO_2_-NPs did not induce any changes of VEGFA expression, we focused on the analysis of the vessels’ neoformation only using CeO_2_-NPs under stress conditions. Retinal vasculature was investigated through whole mounted retinas stained with Isolectin B4 (Figure 3D). An intravitreal injection of CeO_2_-NPs before LD (CeO_2_-NPs-LD group) inhibited neovascularization of the deep retinal plexus; in fact, the vessels’ percentage area was similar to CTRL and was significantly reduced if compared to the LD group (Figure 3B). Additionally, the number of tufts of neovascularization was significantly decreased in CeO_2_-NPs-LD group compared to the LD group, but it was significantly increased compared to the CTRL group (Figure 3C). Representative confocal images of the deep retinal plexus from all experimental groups are reported in Figure 3D.

Notably, CeO_2_-NPs also inhibit the sprouting of choroidal vessels into the photoreceptor layer. As shown in Figure 3E, in the LD retina, the photoreceptors’ outer segments (OS) are lacking and the choroid and neuroretina become closer, favoring the invasion of choroidal vessels into the photoreceptor layer (Figure 3E, white arrows). This was clearly prevented by CeO_2_-NPs, which, conversely, allowed the maintenance of a correct retinal structure and inhibited choroidal neovascularization. This is in agreement with the already demonstrated protection of the RPE by CeO_2_-NPs in the same model [14], reflecting, in turn, the presence of a healthy BRB.

Altogether, the results of the in vivo experiment in the AMD model suggest that CeO_2_-NPs are effective in preventing retinal vascular alterations. This protective effect could be mediated by different mechanisms of action, one of which is represented by VEGFA inhibition.

### 3.2. CeO_2_-NPs Counteract H_2_O_2_-Induced Oxidative Stress in ARPE-19

It is known that H_2_O_2_ induces oxidative stress and apoptosis in different human cells, leading to the overproduction of ROS, which, in turn, causes cellular damage by altering the oxidants-antioxidants equilibrium system [28,29]. Such alterations also occur in AMD [6]. Therefore, to better understand the mechanisms of protection of CeO_2_-NPs observed in vivo, we performed experiments in an in vitro model of oxidative stress already used in our previously published paper [14]. Based on the results found in the literature, demonstrating the efficacy of CeO_2_-NPs at a concentration ranging from about 0.01 mM up to 0.5 mM in vitro, we used a concentration of 0.1 mM, a dose tenfold lower than that used in our in vivo experiment [30,31,32].

**Figure 4:**
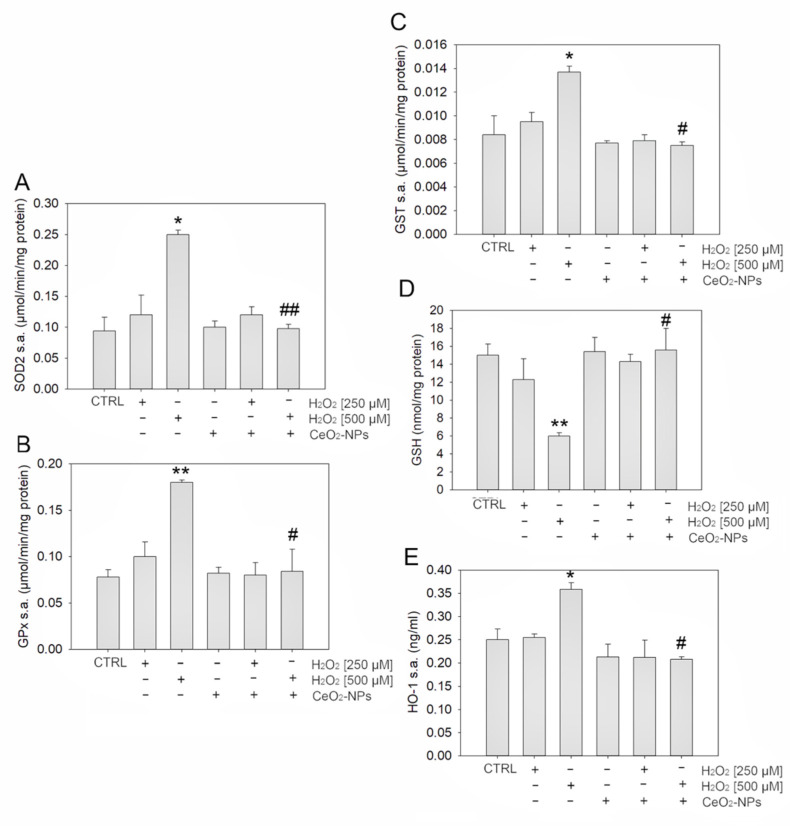
Antioxidant effects of CeO_2_-NPs on ARPE-19 cells treated with H_2_O_2_. The graphs show the enzyme specific activity of SOD 2 (**A**), GPx (**B**), and GST (**C**), as well as GSH (**D**) and HO-1 (**E**) levels. Histograms indicate the means ± SE of three different cultures each of which was tested in triplicate. Statistical analysis was performed by one-way ANOVA followed by Tukey’s test. * *p* < 0.05, ** *p* < 0.01 vs. CTRL, and # *p* < 0.05, ## *p*<0.01 vs. H_2_O_2_.

We first investigated whether 0.1 mM CeO_2_-NPs, added to the medium together with H_2_O_2_, were able to counteract oxidative stress burden and restore the antioxidant defenses altered by H_2_O_2_ treatment in ARPE-19 cells. In particular, we analyzed SOD 2, GPx, and GST activity together with the GSH level, the first-line antioxidant defense, and the level of HO-1, a key regulator of cellular redox homeostasis (Figure 4) [33]. We found that 500 µM H_2_O_2_ induced a significant increase in SOD 2-, GPx-, and GST-specific activities, as well as in the HO-1 level; moreover, this concentration of H_2_O_2_ induced a significant decrease in GSH levels. Conversely, the combined treatment of CeO_2_-NPs with H_2_O_2_ induced the rescue of all the considered markers, allowing ARPE-19 cells to maintain their endogenous antioxidant abilities at the same level of the control (Figure 4).

### 3.3. CeO_2_ NPs Counteract Dicarbonyl Stress in ARPE-19

It is known that oxidative stress can induce lipid peroxidation and glycoxidation reactions, with the consequent abnormal accumulation of intracellular methylglyoxal (MG) [34]. MG, an extremely reactive dicarbonyl compound, is a potent arginine-directed glycating agent and precursor to the major advanced glycation end product (AGE), arginine-derived hydroimidazolone MG-H1 [35]. Excessive MG-derived MG-H1 accumulation, due to MG increased production and/or decreased detoxification by its scavenger enzyme glyoxalase 1 (Glo1), generates a pathological condition called dicarbonyl stress [36].

**Figure 5:**
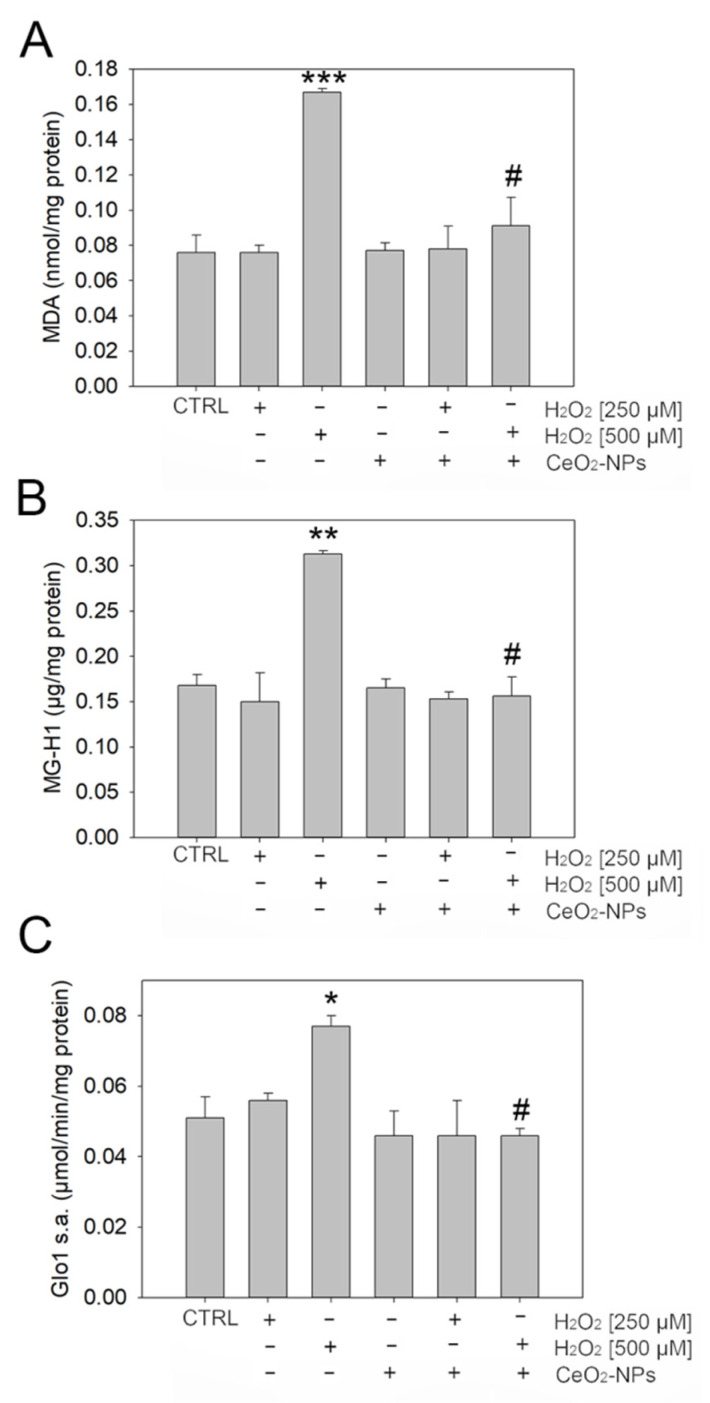
Effect of CeO_2_-NPs (0.1 mM) on dicarbonyl stress in ARPE-19 treated with two concentrations of H_2_O_2_ (250 and 500 µM). (**A**) Malondialdehyde (MDA) levels; (**B**) MG-H1 levels; (**C**) Glo1 specific activity. Histograms indicate the means ±SE of three different cultures, each of which was tested in triplicate. Statistical analysis was performed by one-way ANOVA followed by Tukey’s test * *p* < 0.05 and ** *p* < 0.01, *** *p* < 0.001 vs. control. # *p* < 0.05 vs. H_2_O_2_.

Hence, to investigate whether, in ARPE-19, H_2_O_2_-driven oxidative stress was associated with both lipid peroxidation and MG-dependent dicarbonyl stress, we evaluated the levels of malondialdehyde (MDA), a marker of lipid peroxidation, and MG-H1 together with Glo1 specific activity, to detect dicarbonyl stress. As shown in Figure 5A–C, 500 μM H_2_O_2_ induced an increase of MDA, MG-H1, and Glo1. Again, CeO_2_-NPs were able to rescue the levels of MDA MG-H1 and the specific Glo1 activity, bringing them to the level of CTRL.

### 3.4. Effects of CeO_2_-NPs on ARPE-19 Cells Morphology and Cytoskeleton Organization

**Figure 6:**
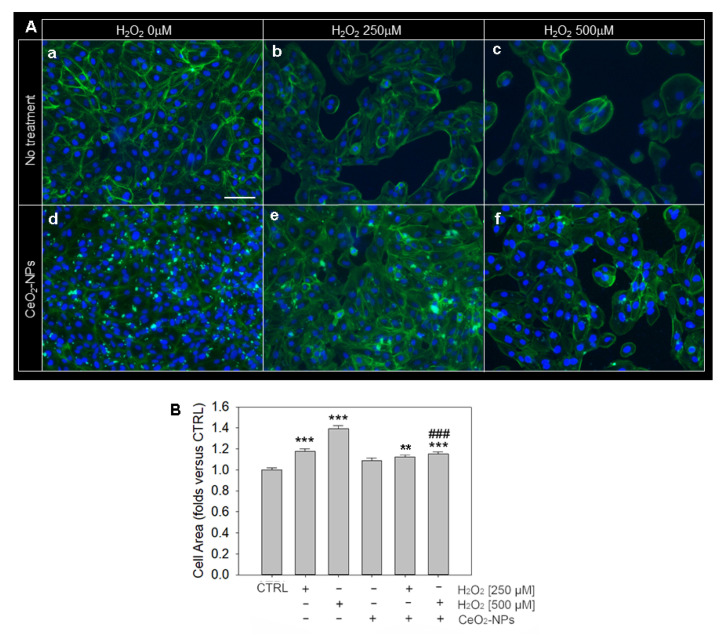
Phalloidin staining of ARPE-19 cells. (**A**) ARPE-19 cells stained with phalloidin (green) and bisbenzimide (blue), which label α-actin and nuclei, respectively. (**a**) Control cells; (**b**) cells exposed to 250 µM H_2_O_2_ and (**c**) 500 µM H_2_O_2_; (**d**) cells treated with 0.1 mM CeO_2_-NPs; (**e**) cells exposed to 250 µM H_2_O_2_ and treated with 0.1 mM CeO_2_-NPs; (**f**) cells exposed to 500 µM H_2_O_2_ and treated with CeO_2_-NPs 0.1 mM. Scale bar: 100 µm. (**B**) The graph shows the analysis of the ARPE-19 area with six experimental conditions (control, H_2_O_2_ 250 µM, 500 µM, CeO_2_ 0.1 mM, H_2_O_2_ 250 µM/CeO_2_ 0.1 mM, H_2_O_2_ 500 µM/CeO_2_ 0.1 mM). Data are shown as mean ±SE. One-way ANOVA test followed by Tukey’s test was used to perform statistical analysis (*n* = 200). ** *p* < 0.01; *** *p* < 0.001 versus CTRL; ### *p* < 0.001 versus H_2_O_2_.

The in vitro model of oxidative stress was also used to evaluate possible signs of cell damage associated with changes in cell morphology and cytoskeleton organization. For this purpose, we used phalloidin staining, which detects cytoskeletal actin (Figure 6) on ARPE-19 cells upon H_2_O_2_ exposure at 250 µM and 500 µM.

We did not observe evident morphological alterations in the localization of α-actin in all experimental conditions. However, we observed a significant increase in cell area upon H_2_O_2_ exposure compared to the control (Figure 6A,B), suggesting a condition of cell stress and hypertrophy. In particular, the cell area increased as a function of H_2_O_2_ concentration (250 µM H_2_O_2_: 1.2-fold over CTRL; 500 µM H_2_O_2_: 1.4-fold over CTRL). On this basis, we wondered whether CeO_2_-NPs (0.1 mM) would prevent H_2_O_2_-induced cell hypertrophy in our experimental conditions. First, we demonstrated that the treatment with CeO_2_-NPs does not cause a modification of the area of the ARPE-19 compared to the control. The capability of CeO_2_-NPs in counteracting the increase in cell area is most evident at the highest concentration of H_2_O_2_ (500 µM), when ARPE-19 cells are subjected to a more severe stress, as shown in Figure 6B.

### 3.5. CeO_2_-NPs Counteract the Effects Exerted by CM from ARPE-19 Cells on Tubule Formation

**Figure 7:**
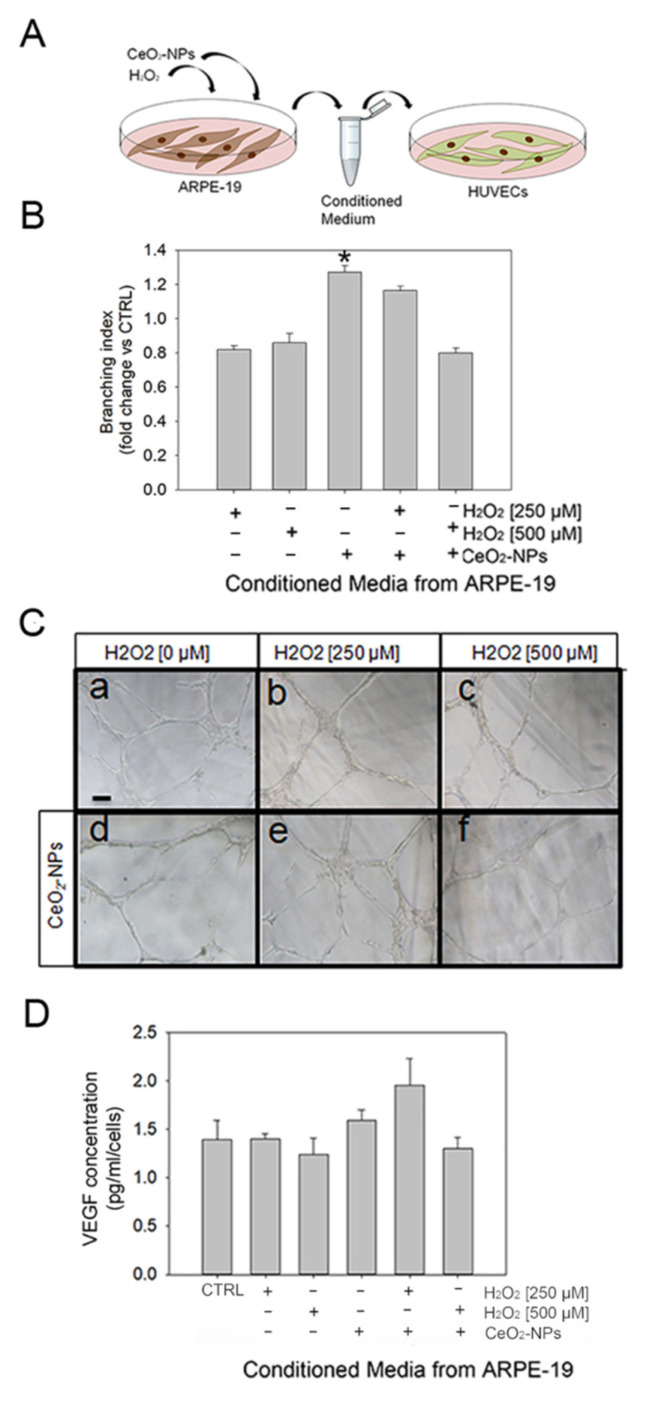
Effects of H_2_O_2_ on tubule formation in vitro. (**A**) Schematic representation of the experimental workflow. (**B**) Histogram shows the effect of CM from ARPE-19 cells exposed to different treatments (H_2_O_2_ at 250 and 500 μM; CeO_2_-NPs at 0.1 mM, and in combination) on the tubule formation of HUVECs. The number of tubules has been measured by counting the number of junctions/area (branching index). (**C**) Representative images of tubule formation of HUVECs exposed to CM from ARPE-19 treated with H_2_O_2_ (250 µM and 500 µM) in the presence or absence of CeO_2_-NPs (0.1 mM) (**a**–**f**); scale bar: 200 mm. (**D**) VEGF determination in CM from ARPE-19 cells treated with H_2_O_2_ (250 µM and 500 µM) in the presence or absence of CeO_2_-NPs (0.1 mM). Statistical analysis: one-way ANOVA followed by Tukey’s test (* *p* < 0.05 vs. CTRL).

To better understand the mechanism by which oxidative stress-induced changes on ARPE-19 could affect angiogenesis in vitro, we investigated whether the conditioned media (CM) from H_2_O_2_-treated ARPE-19 could influence the ability of HUVECs to form tubule-like structures, and whether CeO_2_-NPs could interfere with this response. For this purpose, HUVECs were exposed to CM from ARPE-19 cells treated with H_2_O_2_ with or without CeO_2_-NPs (Figure 7A). We found that CM from ARPE-19 treated with H_2_O_2_ induced a slight but not significant decrease in the number of tubules formed, as demonstrated by the number of junctions per area (branching index), and a significant increase in the number of tubules formed in the presence of CeO_2_-NPs only (Figure 7B,C). However, this trend was reverted in HUVEC in the presence of CM from ARPE-19 treated with H_2_O_2_ in combination with CeO_2_-NPs (Figure 7B,C). VEGF secretion levels showed a similar trend but were not significantly affected by the treatments (Figure 7D).

Although important, these experiments were not conclusive in determining the significance of the effects observed on tubule formation because they a priori exclude the reciprocal interaction between ARPE-19 cells and HUVECs. Moreover, both RPE and retinal endothelial cells are exposed to oxidative stress in vivo; therefore, the eventual direct effect of H_2_O_2_ on HUVECs may influence the crosstalk between the two cell types. On this basis, further experiments were set up. First, HUVECs were exposed to increasing concentrations of H_2_O_2_ to determine the impact on viability (Supplementary Figure S3A). Moreover, HUVECs were subjected to a tubule formation assay when treated with increasing concentrations of H_2_O_2_ (250 µM, 500 µM, 600 µM, 800 µM, 1000 µM, and 1200 µM) (Supplementary Figure S3B).

**Figure 8:**
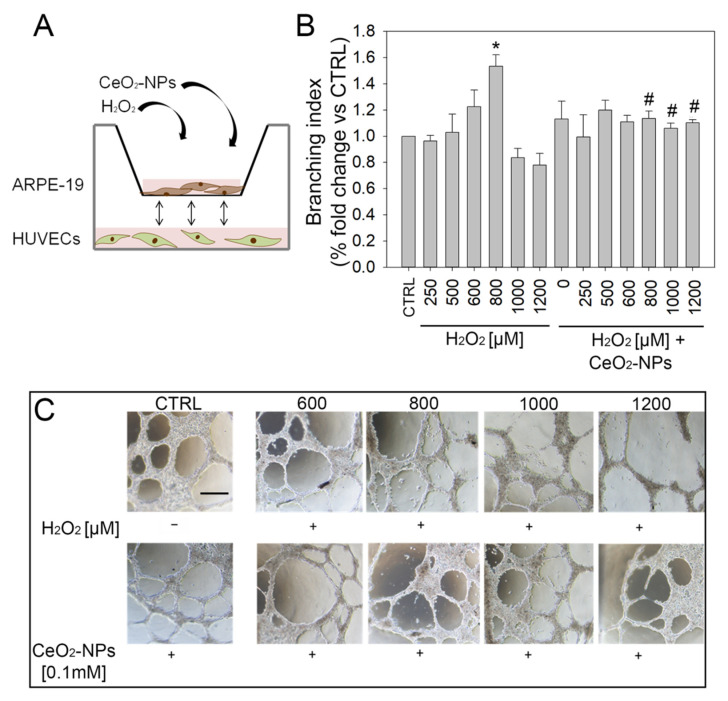
Effects of H_2_O_2_ on a modified co-culture (ARPE-19-HUVECs) tubule formation in vitro. (**A**) Schematic representation of experimental workflow. (**B**) Tubule formation in co-cultured HUVECs with ARPE-19 cells treated with H_2_O_2_ (250, 500, 600, 800, 1000, and 1200 µM) or with H_2_O_2_ plus CeO_2_-NPs (0.1 mM). (**C**) Representative images of tubule formation in different experimental conditions acquired by a Nikon inverted phase contrast microscope; scale bar: 300 mm. Statistical analysis: one-way ANOVA followed by Tukey’s test; * *p* < 0.05 vs. CTRL, # *p* < 0.05 vs. H_2_O_2_.

We observed that, starting from H_2_O_2_ 500 µM, HUVECs viability significantly decreased, thus suggesting a direct effect of H_2_O_2_ (Supplementary Figure S3A). Furthermore, starting from H_2_O_2_ 400 µM, tubule formation was also altered; as shown in the histogram, at 400 µM, an increase of branching index was demonstrated, while at 600 µM of H_2_O_2_, the tubule formation dramatically dropped (Supplementary Figure S3B). These observations confirmed the hypothesized direct effect of H_2_O_2_ on HUVECs. Therefore, a modified tubule formation assay with the co-presence of the two cell types, ARPE-19 and HUVECs (Figure 8A), was performed in order to evaluate the results of the reciprocal interaction between them and to mimic, as close as possible, an in vivo setting.

For this purpose, ARPE-19 cells were treated with various combinations of H_2_O_2_ (250 µM, 500 µM, 600 µM, 800 µM, 1000 µM, and 1200 µM) and 0.1mM CeO_2_-NPs. Then we used ARPE-19-treated cells in co-culture with HUVECs as described in Materials and Methods. The medium was added in each µ-Dish to allow for the exchange of factors between ARPE-19 and HUVECs in response to H_2_O_2_ stress. Our results show that H_2_O_2_ determines a progressive rise of tubules formation with a significant increase when ARPE-19 are treated with H_2_O_2_ 800 µM (400 µM after dilution 1:1 with EGM-2) (Figure 8B). Conversely, at the higher H_2_O_2_ concentrations, 1000 μM (500 µM after dilution with EGM-2 1:1) and 1200 µM (600 μM after dilution 1:1 with EGM-2), tubule-like structures were significantly reduced. Moreover, the cells appeared rounded and dark, suggesting a possible start of apoptosis. CeO_2_-NPs prevented both the H_2_O_2_-induced increase of tubule-like structures observed at 800 μM and H_2_O_2_-reduced tubules observed at 1000 μM, reverting the pro-oxidative effect and/or cytotoxic effect of H_2_O_2_. Thus, CeO_2_-NPs are able to restore the tubule formation ability of HUVECs, co-cultured with ARPE-19 treated with H_2_O_2_ and CeO_2_-NPs, to a level comparable to that of the CTRL (Figure 8B).

## 4. Discussion

CeO_2_-NPs are a new promising nanotechnology for biomedical applications due to their peculiar properties. Indeed, they are synthesized to obtain a non-stoichiometric compound with auto-regenerative radical scavenging activity [13]. Accordingly, CeO_2_-NPs have shown beneficial therapeutic outcomes in multiple models of pathologies characterized by oxidative stress burden. The purpose of this study was to investigate whether CeO_2_-NPs are able to counteract retinal neovascularization under stress conditions, the main feature of wet AMD. To this purpose, CeO_2_-NPs were tested in the light damage (LD) animal model of AMD, which recapitulates some aspects of wet AMD. Moreover, since it is known that RPE dysfunction plays a fundamental role in retinal neovascularization [37], we focused our attention on the RPE–endothelial cells interaction in vitro. Altogether, the results of our study add important elements to the knowledge of the mechanisms by which CeO_2_-NPs can counteract retinal degeneration as it occurs in AMD.

The main findings of the study are discussed below in detail.

### 4.1. CeO_2_-NPs Inhibit Retinal Neovascularization and VEGFA Up-Regulation in the Light Damage Model of AMD

To investigate whether CeO_2_-NPs were able to counteract pathological neovascularization, as it occurs in wet AMD, the in vivo studies were conducted on the LD model. Indeed, light damage has recently emerged as a suitable method to induce retinal degenerative features that are characteristic of wet AMD [19]. In particular, in our previous studies, we demonstrated that the exposure of albino rats to intense light for 24 h causes VEGF up-regulation, new vessels formation and their infiltration into the photoreceptor layer [19]. In addition to neovascularization, other important aspects of AMD are mimicked by the LD model: oxidative stress, inflammation, microglia activation [38], RPE degeneration [14], the accumulation of auto-fluorescent debris [18], and photoreceptor death [15,38]. Moreover, there is a specific area of the rat retina called “hotspot” where the first degenerative events are triggered, and this area was used to perform the in vivo analysis, as shown in Figure 2. On this basis, the LD model represents a suitable method to study possible therapeutic strategies for AMD. In the presence of LD, we previously demonstrated that CeO_2_-NPs are able to maintain a physiological retinal function, prevent photoreceptors death, contrast inflammation and gliosis, and preserve the integrity of RPE cells [14]. Starting from these results, in this study we wondered whether the capability of CeO_2_-NPs to protect RPE could also have relevance in the regulation of angiogenesis.

Notably, here we demonstrated that CeO_2_-NPs’ administration was able to reduce the levels of VEGFA and retinal or choroidal neovascularization in the LD model. Previous studies showed a similar effect in a transgenic mouse model of AMD (Vldlr^−/−^mice) [39,40], and in the laser-induced CNV mouse model of AMD [41]. Following up on such studies, here we tested for the first time the anti-neovascular effects of CeO_2_-NPs in an in vivo model of AMD induced by a photo-oxidative damage. Based on the key role played by an injured RPE in promoting pathological neovascularization in AMD [3], the protective effect of CeO_2_-NPs observed in this study may be mediated by their ability to maintain a healthy RPE. This hypothesis is supported by previous evidence, reporting that intravitreally injected CeO_2_-NPs are found localized in RPE cells [14,42] and prevent their dysfunction and cell death in the LD model. This is particularly relevant, since the RPE, together with the choroidal and retinal vasculature, constitutes the BRB, and allows the maintenance of a controlled microenvironment and of a selective barrier for the diffusion of substances towards the retina. In this context, the oxidative stress plays a major role in BRB breakdown, as observed in AMD, and is the basis of a vicious cycle characterized by the accumulation of toxic metabolites and free radicals, with increasing oxidative stress burden and progression from early to late stages of the disease [6]. Moreover, oxidative stress is a major inducer of VEGF overexpression and, subsequently, of neovascularization. Therefore, based on the well-established antioxidant properties of CeO_2_-NPs, the anti-neovascularization effects of CeO_2_-NPs are likely to be mediated by their antioxidant activity, which in turn might inhibit neo-angiogenesis. In addition to the protection of RPE cells, CeO_2_-NPs may exert their anti-neovascular effect through the inhibition of microglia activation in the retina. In fact, microglial activation and migration from the inner to the outer retina is an additional process that occurs in AMD, as well as in the degenerative processes induced by LD in our model. Notably, increasing evidence indicates that microglia activation may be involved in VEGF up-regulation and contribute to retinal neovascularization. In our previous studies, we demonstrated that CeO_2_-NPs are able to prevent microglia reactivity in the LD model, and this may, in turn, prevent neovascularization. This is in agreement with a previous study in which CeO_2_-NPs inhibited retinal neovascularization and the up-regulation of inflammatory factors in Vldlr^−/−^ mice [39].

From a translational point of view, the administration of CeO_2_-NPs would be advantageous for patients suffering from wet AMD. First, previous studies already demonstrated that intravitreal injections of CeO_2_-NPs are safe and are not cytotoxic for the retina [43,44]. Secondly, but not less importantly, CeO_2_-NPs are retained in the retina for at least one year following a single intravitreal injection [44]. Notably, due to their auto-regenerative ROS scavenging properties [13], they also maintain their activity without exhaustion after a single administration. This is of particular relevance, since the success of a possible clinical translation of CeO_2_-NPs would overcome one of the major problems of current anti-VEGF therapies, which require multiple intravitreal injections over time, and therefore the occurrence of several side effects, as well as patient discomfort.

### 4.2. CeO_2_-NPs Protect ARPE-19 Cells Restoring Antioxidant Defenses

The protective effects of CeO_2_-NPs in counteracting oxidative stress burden and restoring the antioxidant defenses altered by H_2_O_2_ treatment in ARPE-19 cells was demonstrated in an in vitro model. The effect of H_2_O_2_ in inducing the overproduction of ROS has been demonstrated in several human cell lines, which, in turn, causes cellular damage by altering the oxidants-antioxidants equilibrium system [28,29,45]. The RPE has a high metabolic rate and consequently is subjected to the action of ROS, which induce DNA, lipids, and protein damage. For this reason, it is not surprising that RPE and retinal cells possess many enzymatic and non-enzymatic antioxidant defenses. Among them, GSH is considered the main regulator of intracellular redox homeostasis by acting directly through the removal of free radicals and indirectly by becoming a substrate of enzymes, such as GPx or GST. GSTs have an important role in cell detoxification by catalyzing the conjugation of GSH to endogenous or exogenous oxidative stress products, and by contributing to their solubilization and excretion from the cell [46]. Furthermore, GSH participates in what is called “the first line of defense” against oxidative stress [47].

Accordingly, our results show that H_2_O_2_ 500 µM determines an alteration of cellular redox homeostasis, inducing a significant increase in SOD 2-, GPx-, and GST-specific activities. In line with this, the HO-1 level, a known key regulator of cellular redox homeostasis, was also significantly increased in H_2_O_2_-stimulated ARPE-19 cells [33]. More importantly, at these concentrations, CeO_2_-NPs restored cellular physiological conditions, allowing ARPE-19 cells to maintain their endogenous antioxidant abilities at the same level of the control. This is in agreement with the known antioxidant properties of CeO_2_-NPs [48].

It is also known that ROS accumulation under oxidative stress can induce lipid peroxidation and glycoxidation reactions, both leading to an abnormal accumulation of intracellular methylglyoxal (MG) [34]. MG, an extremely highly reactive dicarbonyl compound, is the most potent arginine-directed glycating agent in humans and it is the precursor to the major advanced glycation end product (AGE), named arginine-derived hydroimidazolone MG-H1 [35]. MG, and consequently MG-H1 levels, are controlled by the main MG scavenger enzyme, glyoxalase 1 (Glo1). The accumulation of MG-derived MG-H1, as a result of increased MG formation and/or Glo1 dysfunction, generates a pathological state, called dicarbonyl stress [36]. Dicarbonyl stress has been associated with several age-related diseases, such as neurological disorders, cancer, diabetes and diabetes-associated complications, and AMD, as a consequence of their ability to modify molecules and to form nondegradable aggregates, which accumulate into the cells and impair normal cellular and tissue functions [49,50]. In line with these results, we found that H_2_O_2_ 500 μM induced lipid peroxidation, as indicated by the observed marked increase in MDA levels, and MG-H1 accumulation. The observed increase in Glo1 functionality very likely suggests the incapacity of this MG metabolizing enzyme to cope with H_2_O_2_-dependent MG-H1 production, thus further favoring MG-H1-dependent dicarbonyl accumulation and dicarbonyl stress onset. As expected, CeO_2_-NPs were able to rescue the levels of MDA, MG-H1, and Glo1 restoring physiological levels.

Morphological changes and hypertrophy are cellular alterations often observed in H_2_O_2_ treated cells [51]. These parameters were also measured in our in vitro model and, in compliance with the literature, our results demonstrated that the cell area increased as a function of H_2_O_2_ concentration, indicating a condition of cellular stress and hypertrophy.

Collectively, these results confirmed that, in an in vitro model, CeO_2_-NPs are able to revert ROS-dependent effects on human retinal pigment epithelial (ARPE-19) cells.

### 4.3. CeO_2_-NPs Are Able to Restore the Tubule Formation Ability of HUVECs, Protecting ARPE-19 Cells and HUVECs from Oxidative Stress-Induced Cell Damage

In vitro, we investigated angiogenesis in relation to RPE–endothelial cells interaction under oxidative stress and in the presence or absence of CeO_2_-NPs. To this end, we performed two experimental approaches: (i) treatment of HUVECs with CM from ARPE-19 cells, and (ii) co-culture of ARPE-19 and HUVECs.

It is well-known that ROS-mediated oxidative stress induces dysfunction of RPE cells, leading to increased VEGF release, resulting in the undesirable CNV in AMD [52]. ROS generated during cell respiration and metabolism are primary mediators regulating various cellular biological processes. In particular, H_2_O_2_ is one of the essential ROS, which can activate several biological processes and a modest increase, and fine regulation of this molecule is essential for the maintenance of vascular homeostasis [53]. In addition, H_2_O_2_, is also able to inhibit angiogenesis as demonstrated in several papers in vitro and in vivo, depending on its concentration [54,55,56].

To verify whether the oxidative stress-induced RPE dysfunction could be responsible for neovascularization, as it occurs in wet AMD, and whether it might be inhibited by CeO_2_-NPs, we tried to reproduce this condition by using CM derived from ARPE-19 cells treated with H_2_O_2_ to perform an in vitro tubule formation assay. In this system, H_2_O_2_ induced a slight but not significant decrease in the number of tubules formed. However, this trend seemed to be reverted in HUVEC in the presence of CM from ARPE-19 treated with H_2_O_2_ in combination with CeO_2_-NPs. This kind of assay did not clearly identify the cause of the effects observed on tubule formation in vitro and seemed to be not completely in line with results obtained in vivo and with some literature data reporting that ARPE-19 cells release pro-angiogenic factors when exposed to oxidative insult, leading in turn to angiogenesis [57]. On the other hand, indirect effects of H_2_O_2_ on HUVECs cells have been reported to depend on the cell model and on the concentration and duration of exposure [58,59].

Remarkably, in an in vivo system, both RPE and retinal endothelial cells are exposed to oxidative stress, therefore, the eventual direct and indirect effect of H_2_O_2_ on HUVECs may influence the crosstalk between the two cell types. Thus, the assessment of the potential effect of H_2_O_2_ on neovascular events was performed using an RPE-HUVEC co-culture system in vitro to mimic the effect of oxidative stress both on RPE and endothelial cells.

Through this model, we demonstrated that H_2_O_2_ determined a progressive rise of tubule formation up to a threshold-concentration beyond which, conversely, tubule-like structures were significantly reduced because of dramatic effects on cell viability. Importantly, CeO_2_-NPs were able to revert such detrimental effects by realigning the level of tubule formation to the control for all the tested concentrations of H_2_O_2_.

The obtained results are consistent with our hypothesis; thus, we can suppose that the correct maintenance of retinal vasculature by CeO_2_-NPs also requires the protection of RPE cells from oxidative stress.

## 5. Conclusions

Altogether, our data add new knowledge about the mechanisms through which CeO_2_-NPs are able to mediate retinal protection. We demonstrated, for the first time, that CeO_2_-NPs counteract retinal neovascular alterations induced by oxidative damage in vitro and in vivo. Therefore, the results of the present study indicate that CeO_2_-NPs could be a possible new therapeutic strategy for the treatment of AMD, and further studies in this direction may be useful to achieve this purpose.

## Data Availability

Data is contained within the article or supplementary material.

## Supplementary Materials

The following supporting information can be downloaded at: https://www.mdpi.com/article/10.3390/antiox11061133/s1, Supplementary Figure S1. (**A**) Western blot analysis of VEGFA in CTRL and CeO2-NPs injected healthy animals (CeO2-NPs-CTRL) groups (*n* = 4). The graph shows the mean ± SE of VEGFA (folds vs. CTRL) normalized versus α-Tubulin. Statistical analysis: one-way ANOVA followed by Tukey’s test, ** *p* < 0.01. (**B**) Bands of entire Western blot. For Figure S1A, the third and fourth bands were used as representative. Adobe Photoshop was used to crop the horizontal line of VEGFA and α -Tubulin; Supplementary Figure S2. Bands of entire Western blot. For Figure 3A, the third, fourth, and seventh bands were used as representative. Adobe Photoshop was used to crop the horizontal line of VEGFA and α -Tubulin; Supplementary Figure S3. (**A**–**C**) Dose dependent H_2_O_2_ effects on viability and on tubule formation of HUVECs. (**A**) H_2_O_2_ treatment determined a significant increase in tubule formation ability from 400 μM, as shown by branching index analysis, and then, at a higher concentration, a dramatic reduction in the number of tubules was observed. (Branching index: number of junctions/area) * *p* < 0.05; ** *p* < 0.01; *** *p* < 0.001. Scale bar: 100 μm.
