# Supplementary material for: Antioxidant Properties of Cerium Oxide Nanoparticles Prevent Retinal Neovascular Alterations In Vitro and In Vivo
Source: Antioxidants (Basel). 2022 Jun 9;11(6):1133. doi: 10.3390/antiox11061133 (PMC9220105; doi:10.3390/antiox11061133)
Supplement: Supplementary file 1 [file antioxidants-11-01133-s001.zip › antioxidants-1746052-supplementary.pdf]

Supplementary Figures

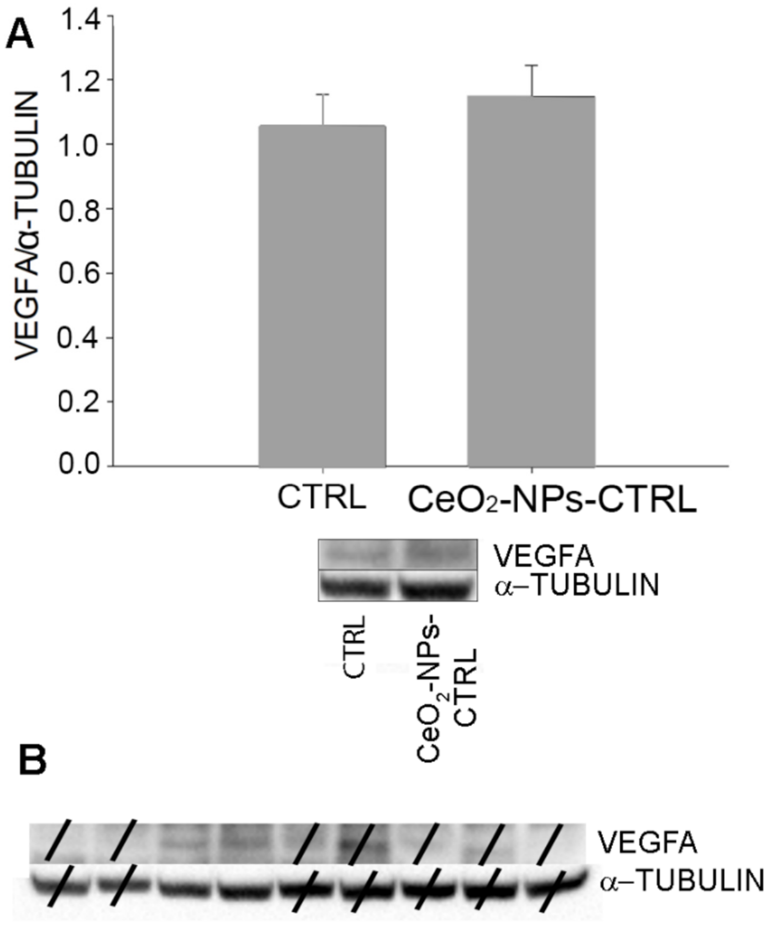

Figure S1. (**A**) Western blot analysis of VEGFA in CTRL and CeO_2-NPs injected healthy animals (CeO_2-NPs-CTRL) groups (n = 4). The graph shows the mean ± S.E. of VEGFA (folds vs CTRL) normalized versus α-Tubulin. Statistical analysis: one-way ANOVA followed by Tukey test, ** $p < 0.01$. (**B**) Bands of entire western blot. For Figure S1A the third and fourth bands were used as representative. Adobe Photoshop was used to crop the horizontal line of VEGFA and -Tubulin.

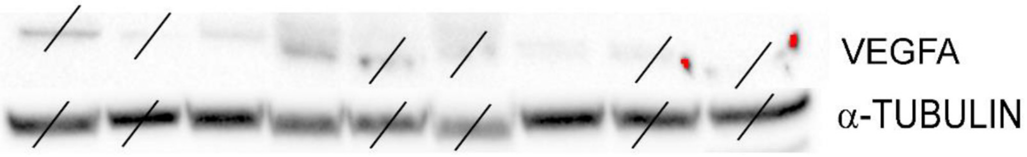

Figure S2. Bands of entire western blot. For the Figure 3A, the third, fourth and seventh bands were used as representative. Adobe Photoshop was used to crop the horizontal line of VEGFA and -Tubulin.

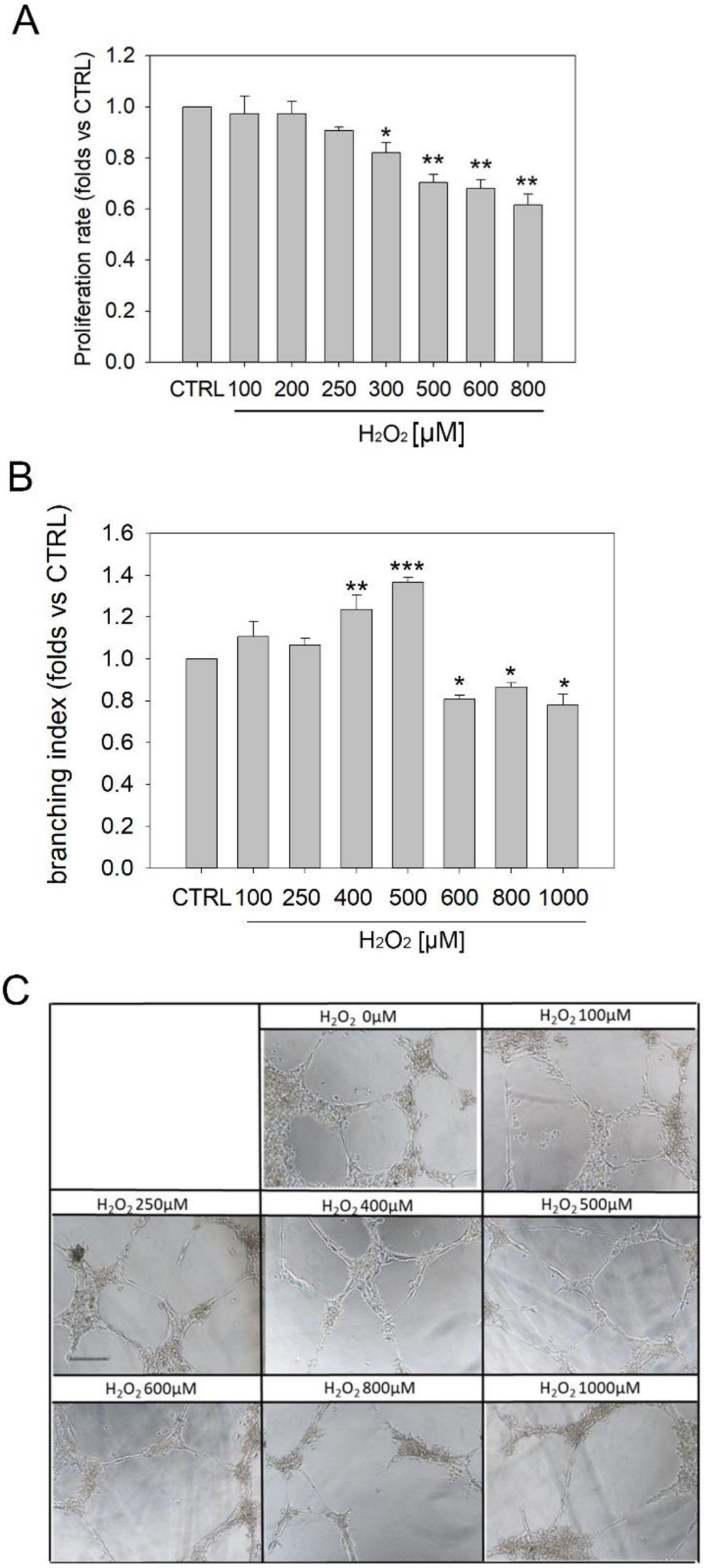

Figure S3. (**A, B, C**) Dose dependent H_2O_2 effects on viability and on tubule formation of HUVECs. (**A**) H_2O_2 treatment determined a significant increase of tubule formation ability from 400 μM, as shown by branching index analysis, and then, at higher concentration, a dramatic reduction of the number of

tubules was observed. (Branching index: number of junction /area) * $p < 0.05$; ** $p < 0.01$; *** $p < 0.001$. Scale bar: 100 μm.
